# Yolk Sac Mesenchymal Progenitor Cells from New World Mice (*Necromys lasiurus*) with Multipotent Differential Potential

**DOI:** 10.1371/journal.pone.0095575

**Published:** 2014-06-11

**Authors:** Phelipe Oliveira Favaron, Andrea Mess, Sônia Elisabete Will, Paulo César Maiorka, Moacir Franco de Oliveira, Maria Angelica Miglino

**Affiliations:** 1 School of Veterinary Medicine and Animal Science, University of Sao Paulo, São Paulo, Brazil; 2 Department of Animal Science, Universidade Federal Rural do Semi-Árido, Mossoró, Rio Grande do Norte, Brazil; Rutgers - New Jersey Medical School, United States of America

## Abstract

Fetal membranes are abundant, ethically acceptable and readily accessible sources of stem cells. In particular, the yolk sac is a source of cell lineages that do not express MHCs and are mainly free from immunological incompatibles when transferred to a recipient. Although data are available especially for hematopoietic stem cells in mice and human, whereas other cell types and species are dramatically underrepresented. Here we studied the nature and differentiation potential of yolk sac derived mesenchymal stem cells from a New World mouse, *Necromys lasiurus*. Explants from mid-gestation were cultured in DMEM-High glucose medium with 10% defined fetal bovine serum. The cells were characterized by standard methods including immunophenotyping by fluorescence and flow cytometry, growth and differentiation potential and tumorigenicity assays. The first adherent cells were observed after 7 days of cell culture and included small, elongated fibroblast-like cells (92.13%) and large, round epithelial-like cells with centrally located nuclei (6.5%). Only the fibroblast-like cells survived the first passages. They were positive to markers for mesenchymal stem cells (Stro-1, CD90, CD105, CD73) and pluripotency (Oct3/4, Nanog) as well as precursors of hematopoietic stem cells (CD117). In differentiation assays, they were classified as a multipotent lineage, because they differentiated into osteogenic, adipogenic, and chondrogenic lineages and, finally, they did not develop tumors. In conclusion, mesenchymal progenitor cells with multipotent differentiation potential and sufficient growth and proliferation abilities were able to be obtained from *Necromys* yolk sacs, therefore, we inferred that these cells may be promising for a wide range of applications in regenerative medicine.

## Introduction

Fetal membranes are abundant, ethically acceptable and readily accessible sources of stem cells in contrast to the common albeit ethically questioned usage of embryonic stem cells [1]–[3]. Compared to stem cells derived from adult tissues, the fetal membrane cell lineages have greater differentiation potential and higher proliferation abilities. Also, they do not express Major Histocompatibility Complex (MHC) surface markers and thus do not cause immunological incompatibilities when transferred to a recipient [4], [5]. Special attention has been drawn to the yolk sac, because this membrane maintains essential functions during early gestation including the presence of pluripotente primordial germ cells that subsequently migrate to the primitive gonads, it contribute to form the midgut as well as the first hematopoietic stem cells that contribute to the development of the vascular system [6]–[9]. In addition, the yolk sac endoderm promotes maternal-fetal exchange [10]. So far, much data on cell differentiation from yolk sac tissues are available for hematopoietic stem cells from mice and humans [6], [8], [11]–[15], whereas other cell types and species are dramatically underrepresented. This is especially the case for mesenchymal stem cells that are regarded multipotent at least. Mesenchymal stem cells from embryos and adults have been recognized for their ability to differentiate *in vitro* and *in vivo* into osteogenic, adipogenic and chondrogenic cell lineages [16], [17]. In cell culture, mesenchymal cells have a fibroblast-like phenotype [18]. According to *The International Society for cellular therapy position statement*, human mesenchymal stem cells can be clearly identified by the characteristic responses to a set of cell type, differentiation and potency markers, i.e. positive response to CD105, CD73 and CD90 and negative response to CD45, CD34, CD14 or CD11b, CD79a, CD19 and HLA [19]. However, the limited proliferation and differentiation potential of adult mesenchymal stem cells [20] and the risk of tumors and ethical issues when using embryonic tissues were the impetus to continue research using fetal membranes. Indeed, the yolk sac was recently reported as a source for mesenchymal stem cells. Such cells from the murine yolk sac differentiate into osteoblasts, chondrocytes and adipocytes [21], [22]. For the human, yolk sac mesenchymal stem cells differentiated into osteogenic, adipogenic and neurogenic lineages [23]–[25], but not into chondrocytes [25]. In addition to the above characteristics, both rodent and human yolk sac derived cells are also immunopositive for further antigens as well as markers for proliferation and pluripotency [22]–[25]. This pattern, and differentiation into mesoderm-derived lineages only are regarded as indications of multipotency. Finally, although there were promising initial results on other species such as dog [26] or sheep [27], a broader sample of species is desired. In particular, it regarding development of animal models to determine whether the mouse reflects a more specialized or more general pattern of rodents. Thus, here we studied the nature and differentiation potential of yolk sac-derived mesenchymal progenitor cells from a New World mice species native to South America, *Necromys lasiurus* (Rodentia, Cricetidae, Sigmodontinae). Explants from mid-gestation yolk sacs were cultured and thereafter cells were characterized by standard methods including immunophenotyping by fluorescence and flow cytometry to identify surface antigen expression, growing and differentiation performance and tumorigenicity assays.

## Materials and Methods

### Ethics Statement

The project was approved by the Ethical Committee of the School of Veterinary Medicine and Animal Science of University of Sao Paulo, Brazil (Protocol number 1766/2009).

### Collection, Cell Culture and Cell Morphology

Yolk sacs of *Necromys lasiurus* from 10 individuals in mid-gestation (15–16 days) were obtained from a breeding group at the University of Mossoró and cultured at the University of Sao Paulo. The samples were plated in 35 mm Petri dishes (Corning, NY, USA) with four media (Table 1). The Petri dishes were incubated at 37°C in a humidified atmosphere of 5% CO_2_. After 24 and 48 hours, no-adherent cells were removed and the medium was replaced. Every 3 days, 70% of the medium was replaced and at 80% of confluence, the cells were harvested with 0.25% trypsin solution (Invitrogen, Carlsbad, CA, USA) and replaced in 25 cm^2^ and 75 cm^2^ flasks (Corning, NY, USA). Progenitor cells from the yolk sac were analyzed morphologically every 3 days, using an inverted microscope (NIKON ECLIPSE TS-100) and the size and format of the cell populations were also evaluated by flow cytometry (FACSCalibur, BD, San Jose, California, USA). The immunophenotype characterization of cells was done on passage four.

**Table 1:** Culture media used to isolate the progenitor yolk sac stem cells from *Necromys lasiurus* (Rodentia, Cricetidae).

Medium	Results
DMEM-High Glucose (LGC Biotecnologia, Cotia, Sao Paulo,Brazil), 10% FBS characterized (HyClone, Thermo Scientific),1% antibiotic solution (Penicilin G 10.00 U mL, 25 mg mL,Streptomycin 10.000 mg mL), 1% l-glutamine 200 Mm, and1% non-essential amino acids. All from Invitrogen, Carlsbad,CA, USA.	Few adhesion onplates, followed by cell death
DMEM-High Glucose (LGC Biotecnologia, Cotia, Sao Paulo,Brazil), 10% FBS defined (HyClone, Thermo Scientific), 1%antibiotic solution (Penicilin G 10.00 U mL, 25 mg mL,Streptomycin 10.000 mg mL), 1% l-glutamine 200 Mm, and1% non-essential amino acids. All from Invitrogen, Carlsbad,CA, USA.	Satisfactorycharacteristics ofgrowth and celladhesion on theplates
DMEM/HAM’S-F12 (1∶1) (LGC Biotecnologia, Cotia, Sao Paulo,Brazil), 10% FBS characterized, 1% antibiotic solution (Penicilin G10.00 U mL, 25 mg mL, Streptomycin 10.000 mg mL), 1% l-glutamine 200 Mm, and 1% non-essential amino acids. All fromInvitrogen, Carlsbad, CA, USA. 1% antibiotic solution (Penicilin G10.00 U mL, 25 mg mL, Streptomycin 10.000 mg mL), 1% l-glutamine 200 Mm, and 1% non-essential amino acids. All fromInvitrogen, Carlsbad, CA, USA.	Few adhesion onplates, followed bycell death
DMEM/HAM’S-F12 (1∶1) (LGC Biotecnologia, Cotia, Sao Paulo,Brazil), 10% FBS defined, 1% antibiotic solution (Penicilin G10.00 U mL, 25 mg mL, Streptomycin 10.000 mg mL), 1% l-glutamine 200 Mm, and 1% non-essential amino acids. All fromInvitrogen, Carlsbad, CA, USA.	Satisfactory celladhesion butmoderate growth

### Colorimetric Assay (MTT)

The colorimetric [3-(4,5- dimethylthiazol-2-yl) – 2,5-diphenyltetrazolium bromide (MTT)] assay was performed according to the protocol previously established [28]. In order to verify cellular proliferation, the analysis was performed every 48 hours over a 12-day. For this, 1×10^5^ cells were plated on 96 wells plates. Basal culture medium was replaced concurrent with analysis. The cells were washed with 100 µL PBS/well; then, 100 µl of MTT solution [1 mL of MTT (5 mg/mL) in 10 mL of culture medium, and 1% of fetal bovine serum] was added and maintained at 37°C for 3 hours. Thereafter, the formazan was precipited and solubilized in 50 µl of dymethyl sulfoxide – DMSO (Sigma-Aldrich, St. Louis, Mo. USA). The analysis was performed in a spectrophotometer (M-Quant – Bio Tek Instruments, Winooski, VT, USA). The results were analyzed by the GraphPad program (GraphPad Software, La Jolla, CA, USA).

### Immunophenotyping by Fluorescence

On passage 4, the yolk sac cells (1×10^4^) were grown on coverslips in a basal culture medium. After 48 hours, cells were washed twice in tris-buffered saline (20 mM Tris-HCl pH 7.4 in 0.15 M NaCl, and 0.05% Tween-20) and fixed for 24 hours in 4% paraformaldehyde following permeabilization with 0.1% Triton X-100 (Sigma, St. Louis, Mo. USA). After blocking with 5% bovine serum albumin, cells were incubated overnight at 4°C with the primary antibodies in bovine serum albumin. Primary antibodies were cytokeratin 18 (RGE53, sc-32329, Santa Cruz Biotechnology, Inc, Europe), vimentin (0.N.602, sc-73259, Santa Cruz Biotechnology), Stro-1 (sc-47733, Santa Cruz Biotechnology), and β-tubulin (2146, Cell Signaling Technology, Danvers, MA, USA). After washing the primary antibodies three times in tris-buffered saline, the fluorescein isothiocyanate conjugated secondary antibody was added and incubated at room temperature for 1 hour. The secondary antibodies were anti-goat IgG, anti-rabbit IgG (DakoCytomation, CA, USA), and anti-mouse IgG (Chemicon International, Temecula, CA, USA). The slides were mounted using Vectashield with DAPI (H-1200, Vector Laboratories, Inc., Burlingame, CA, USA). Digital slides were acquired using a fluorescence microscopy LSM 510 (Carl Zeiss Microscopy, Jena, Germany). For negative controls, an IgG (Clone Ci4, Merck Millipore, Billerica, USA) was substituted for primary antibody.

### Immunophenotyping by Flow Cytometry

Yolk sac cells on passage 4 were re-suspended in FACS (fluorescence-activated cell sorting) buffer, and the concentration adjusted to 10^5^ cells/mL. For intracytoplasmic and nuclear markers, cells were permeabilized with 5 µl 0.1% Triton X-100 for 30 minutes prior to incubation with specific primary antibodies (concentration of 1∶100): Oct 3/4 (C-10, sc5279), Nanog (n-17, sc30331), cytokeratin 18 (RGE53, sc-32329), vimentin (0.N.602, sc-73259)), PCNA-3 (clone PC10; sc-56), Stro-1 (sc-47733), CD73 (C-20, sc-14684), CD105 (2Q1707, sc-71042), CD34 (BI-3C5, sc:19621), CD90 (Thy-1, aTHy-1A1) and CD45 (H-230, sc-25590), all from Santa Cruz Biotechnology, as well as VEGF (Clone VG1, M7273, DakoCytomation, CA, USA), β-tubulin (2146, Cell Signaling Technology, Danvers, MA, USA) and CD117 (c-Kit, SCF-Receptor Ab-6, RB-1518-R7, Thermo Scientific, Lab Vision Corporation, Fremont, CA, USA), all for 45 minutes at room temperature. Then, cells were incubated for 2 hours with secondary antibody (anti-mouse FITC, DakoCytomation, Santa Cruz Biotechnology). The analysis was performed using a flow cytometer (FACSCalibur, BD).

### Adipogenic, Osteogenic and Chondrogenic Differentiation Assays

All differentiation assays were performed in triplicate using yolk sac cells on passage 6. For osteogenic and chondrogenic differentiation, 1×10^4^ yolk sac cells were plated in six-well plates with a basal culture medium. After 24 hours, osteogenic differentiation was induced with a medium composed of DMEN-High, 7.5% of fetal bovine serum, 100 µM ascorbate-2-phosphate (Invitrogen, Carlsbad, CA, USA), and 0.1 µM dexamethasone (Sigma, St. Louis, Mo. USA) that was changed every 3 days. After 10 days, 10 mM of β-glycerol phosphate (Sigma, St. Louis, Mo. USA) was added. At day 21, the cells were washed twice in PBS, fixed for 24 hours in 4% paraformaldehyde and stained with Von-Kossa (Invitrogen, Carlsbad, CA, USA) upon exposure to 100 W light for 60 minutes.

For the detection of alkaline phosphatase activity was used the Sigma fast p-Nitrophenyl phosphate tablets (Sigma-Aldrich, St. Louis, Mo. USA). Adipogenic differentiation was induced adding DMEN-High supplemented with 15% of fetal bovine serum, 10 µM dexamethasone (Sigma, St. Louis, Mo. USA), 100 mM indomethacin (Sigma, St. Louis, Mo. USA), 10 µg/mL insulin, 10.000 ug/mL antibiotics (Sigma, St. Louis, Mo. USA), and 0.5 mM 1-methyl-3-isobutyxanthine (Sigma, St. Louis, Mo. USA). The medium was changed every 3 days. At day 21, the cells were washed twice in PBS and fixed for 24 hours in 4% paraformaldehyde. To detect intracellular lipid accumulations, cells were stained using Sudan II (SC-215923, Santa Cruz, CA, USA) for 5 minutes, washed in 70% alcohol, and in Harris haematoxilin for 2 minutes. The chondrogenic differentiation was done according to protocol established by Mambelli et al. [29]. To prepare a pellet culture, 2×10^6^ cells were re-suspended using a commercial medium (STEMPRO Chondrogenesis Differentiation Kit, Invitrogen, Carlsbad, CA, USA). Half of the medium was changed twice per week over 21 days. During the culture interval, cells were maintained at 37°C in a humidified atmosphere with 5% CO_2._ After 21 days, cell aggregates were embedded in paraffin and sectioned at 3–4 µm in an automatic microtome (LEICA, RM2165). Then, slides were stained using Picrosirius and Masson’s trichrome.

### Tumorigenicity Assay in Nude Mice

Progenitor yolk sac cells (n = 1×10^6^ cells) on passage 4 were re-suspended in DPBS solution (Invitrogen) and intramuscular injected into the left limbs of 2 nude mice. The animals were maintained under specific pathogen-free conditions using an Isorack, and fed on sterile food and water *ad libidum* at the IPEN (Nuclear and Energy Research Institute, University of Sao Paulo, Brazil) for 8 weeks. Every week, the animals were clinically examined to identify possible tumor formation. Then, the animals were euthanazied following the principles of the Ethical Committee of the School of Veterinary Medicine and Animal Science and samples from the biceps, liver, lung, kidney, and abdominal adipose tissue were collected and fixed in 4% paraformaldehyde. Tissues for histopathology were embedded in paraffin and sectioned at 5 µm, stained with haematoxylin and eosin (HE) and investigated with an Olympus microscope (CX 31 RBSFA).

## Results

### Isolation and Growth Characteristics of the Yolk Sac Cells

**Figure 1:**
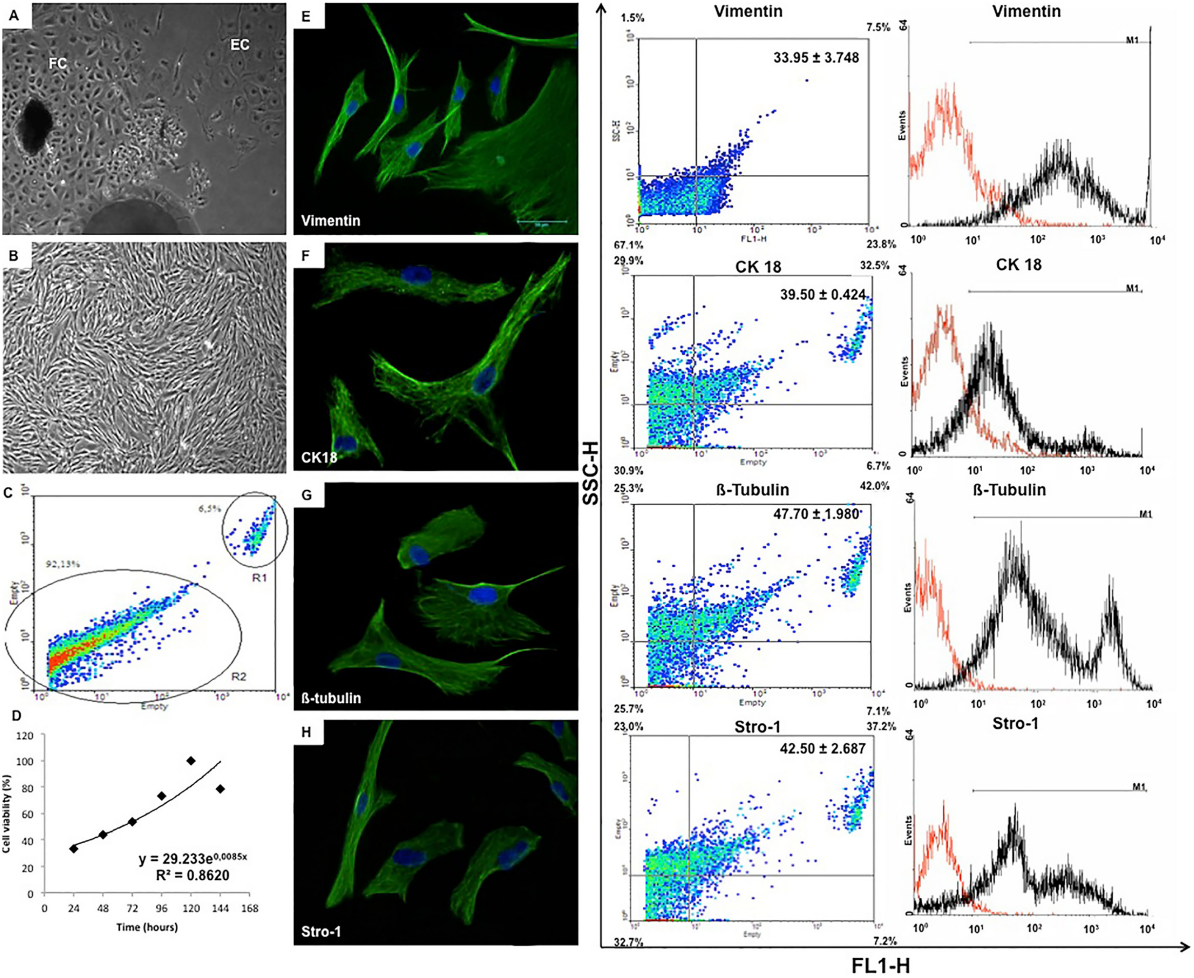
Characteristics of morphology and growth of the cell culture derived from *Necromys lasiurus* yolk sac at mid-gestation and expression of mesenchymal markers by immunohistochemistry and flow cytometry. (**A**): The yolk sac primary culture was composed by fibroblast-like (FC) and epithelial-like (EC) cells. After freezing, only undifferentiated fibroblast-like cells were observed in the culture (**B**). In **C**: Overall, 92.13% of the primary culture was composed by fibroblast-like cells, whereas epithelial-like cells represented 6.5% of the culture. (**D**): According to the MTT assay, the yolk cells had progressive growth until day 5, which was followed by a decrease in cell viability on day 6. On passage 4, 33.95% of the cells expressed vimentin (**E**), 39.5% CK 18 (**F**), 47.7% β-tubulin (**G**), and 42.5% Stro-1 (**H**). In the histograms, positive cells were represented in black and negative in red.

After tested four culture media (Table 1), the best results in regard to cell growth and proliferation were obtained using a combination of DMEM-High glucose medium supplemented with 10% fetal bovine serum defined. Thus, this medium was chosen to perform the experiments. In the primary cell culture the first adherent cells were observed 7 days after the explants were plated. They included two types of cells: fibroblast-like cells were small and elongated and had reduced cytoplasm, whereas epithelial-like cells were large and rounded with sparse cytoplasm (Figure 1A). Both types had centrally located nuclei. Only the fibroblast-like cells survived continued cell passages and application of freezing assays; still showing satisfactory growth potential and uniformity (Figure 1B). In addition, fibroblasts were much more abundant in the cultures than epithelial-like cells (92.1% versus 6.5%; Figure 1C). They were confluent in the plates and maintained the above-described morphology. In order to understand the cellular proliferation by the colorimetric assay, the 1×10^5^ fibroblast-like cells had progressive growth until day 5. After this, was observed a decrease of 22% in cell viability (Figure 1D).

Usually, the yolk sac cells especially the fibroblast-like cells remained growing satisfactorily until passage 4 where the culture was more homogeneous than the previous passages. For this reason, the experiments were performed on passages 4 and 6.

### Immunophenotype Cell Characterization

**Figure 2:**
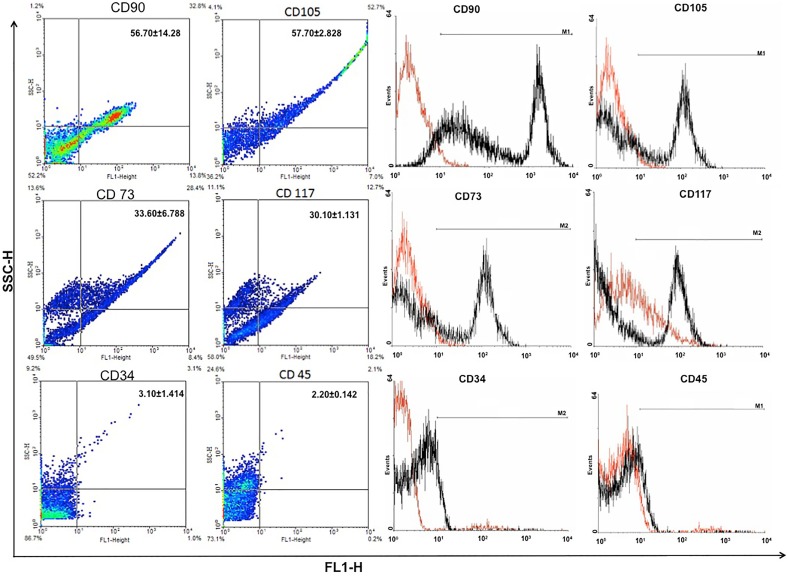
Expression (immunohistochemistry and flow cytometry) of mesenchymal and hematopoietic markers on progenitor derived cells from *Necromys lasiurus* yolk sac at mid-gestation on passage 4. Cells significantly expressed the mesenchymal markers CD90 (56.7%), CD105 (57.7%), CD73 (33.6%) and they also expressed CD117 (30.1%), a specific marker for hematopoietic precursor cells. Neither CD34 nor CD45, specific markers for hematopoietic cells were expressed by the progenitor yolk sac cells. In the histograms, positive cells were represented in black and negative in red.

**Figure 3:**
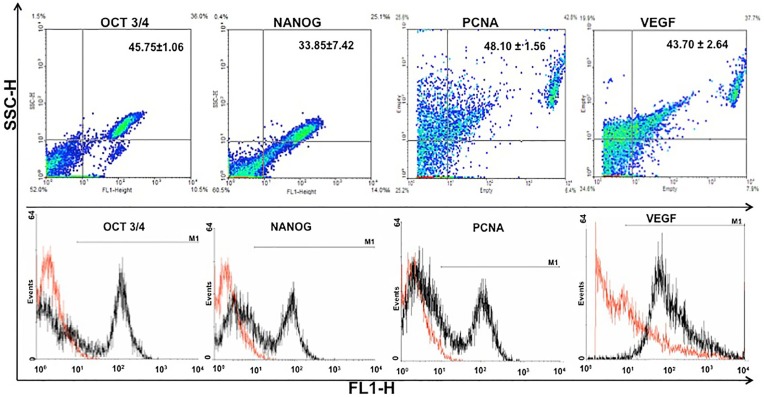
Immunophenotypic characterization (flow cytometry) of progenitor derived cells from *Necromys lasiurus* yolk sac at mid-gestation on passage 4. Totally, 45.75% of the cells expressed Oct ¾ and 33.85% Nanog, both markers of pluripotency. In addition, they expressed VEGF (48.1%) and PCNA-3 (43.70%). Positive cells were represented in the histograms in black and negative in red.

The expression of specific cell types, proliferation and differentiation markers were analyzed by a combination of immunofluorescence and flow cytometry using cells on passage four (Figures 1E–H, 2,3). In both methods, fibroblast-like cells were immunopositive for vimentin (Figure 1E: 33.95±3.75%) and cytokeratin-18 (Figure 1F: 39.50±0.42%), indicating the presence of intermediate filaments, and to β-tubulin (specific for microtubules; Figure 1G: 47.70±1.98%). Also the cells had an immunopositive reaction for Stro-1 (Figure 1H: 42.50±2.69%), and markers of mesenchymal stem cells such as CD90 (56.70±14.28%), CD105 (57.70±2.83%) and CD73 (33.60±6.79%) as well as to CD117, an antibody for hematopoietic precursor cells (30.10±1.13%) (Figure 2). In contrast, the cells did not have significant expression of hematopoietic cells markers such as CD34 (3.10±1.14%) or CD45 (2.20±0.14%). Pluripotency was indicated by expression of Oct3/4 (45.75±1.06%) and Nanog (33.85±7.42%). Finally, positive response was recorded for VEGF (48.10±1.56%) and PCNA-3 (43.70±2.64%), the former indicating an association to the vasculature system and the latter a high percentage of proliferative cells in the cell culture (Figure 3).

### Differentiation Ability

**Figure 4:**
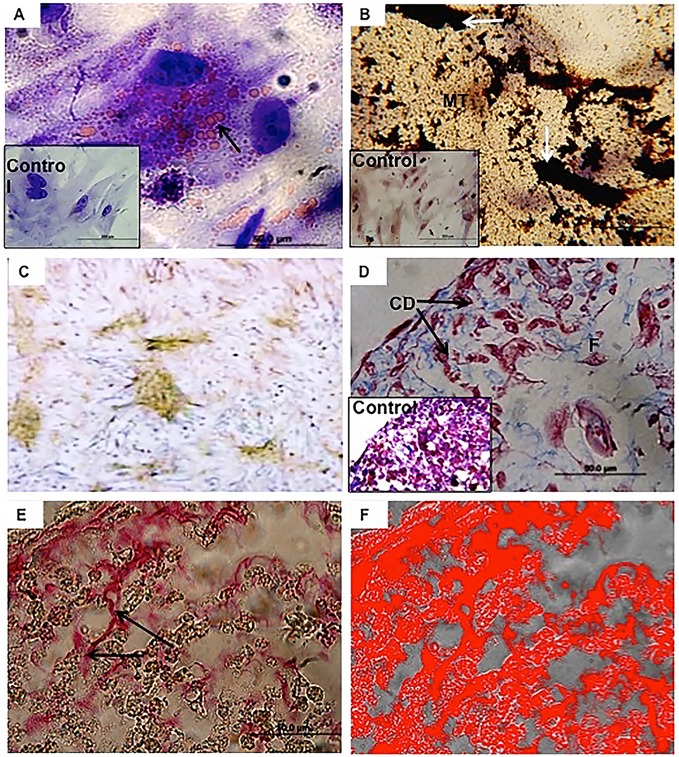
Differentiation assays on passage 6. (**A**): Adipogenic differentiation. The adipocytes cells were organized in groups. The nucleus was located at the periphery of the cytoplasm. Note the intracellular lipid accumulations in the cytoplasm (arrow). Sudan II staining. (**B and C**): Osteogenic differentiation. The cells were polygonal and had a mineralized extracellular matrix (MT) with very condensed areas (arrows). Stained using Von-Kossa. In (**C**): Osteogenic cells showing intracellular phosphatase alkaline activity. (**D, E and F**): Chondrogenic differentiation. In **D**: The parenchymal cells were round with regions similar to condrocyte lacunae (arrow). Stained with Masson’s trichrome. In **E**: Collage fibers were present in the matrix (arrows). In **F**: Collagen occupied 42.3% of the total area of the section.

On passage 6, the yolk sac derived cells were induced to adipogenic, osteogenic, and chondrogenic differentiation by applying specific media routinely used for mesenchymal stem cell differentiation. After 21 days, cells differentiated into the three lineages. Cells with adipocyte morphology were organized in small groups. Their nuclei were displaced and closely attached to the cell membranes. Intracellular lipid accumulations were observed in the cytoplasm after Sudan II staining (Figure 4A). Osteogenic differentiation was confirmed with Von-Kossa staining and by detection of alkaline phosphatase activity. Then the cells were polygonal and had a mineralized extracellular matrix (Figure 4B), and they were able to produce alkaline phosphatase enzyme (Figure 4C). Under chondrogenic differentiation, a suspended pellet formation occurred. Based on Masson trichrome staining, the cells with condrocyte lacunae were frequent (Figure 4D). The matrix was composed of extended collagen areas that were rich in collage fibers (Figure 4E). Using Picrosirius staining under polarized light, collagen areas were quantified (Image J Program) to occupy 42.3% of the total sample (Figure 4F), whereas the control cells without treatment remained their undifferentiated morphology.

### Tumorigenicity of the Yolk Sac Cells

**Figure 5:**
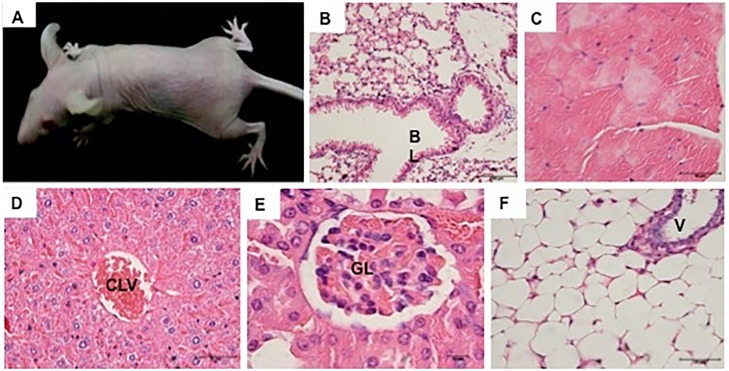
Analysis of the nude mice after 60 days after injection of the yolk sac progenitor cells on passage 4. (**A**): Macroscopic view of the nude mice demonstrating the absence of tumor formation. (**B–F**): Histopathological sections of lung (**B**), skeletal muscle of the left limbs (**C**), liver (**D**), kidney (**E**), and abdominal adipose tissue (**F**), showing the structural integrity and normal cellular arrangement of all analyzed organs.

Immunosuppressed or nude mice did not have any indication of tumor formation within 8 weeks after injection of the yolk sac-derived mesenchymal progenitor cells, neither macroscopically (Figure 5A) nor histopathologically in samples of lung (Figure 5B), skeletal musculature (Figure 5C), liver (Figure 5D), kidney (Figure 5E), and adipose tissue of the abdominal region (Figure 5F).

## Discussion

Although only a few taxa have been investigated in that regard, former studies suggested that the yolk sac is a promising source for mesenchymal progenitor cells [25], [26], [30]. As typical for cells derived from fetal membranes, they do not express MHC on the surface and do not cause immunological incompatibilities usually associated with adult stem cells. In addition, extraembryonic tissues are ethically less problematic to use than parts of the embryo proper. The current results in a species of New World mice (*Necromys lasiurus*) supported these views. We were able to isolate, culture and characterize mesenchymal progenitor cells with defining reactions to immunological surface markers for cell type, differentiation status and proliferation. In addition, the cells had sufficient growth parameters and differentiated into osteogenic, adipogenic and chondrogenic lineages without developing tumors. Thus, the typical characteristics of mesenchymal stems cells from the mouse yolk sac seemed to be widespread among rodents or even larger clades of placental mammals including the human.

From the four tested culture media, the DMEM-High Glucose medium supplemented with defined fetal bovine serum had the best results regarding to cell adhesion, growth and maintenance of the undifferentiated morphology of the fibroblast-like cells during the passages. Interestingly, DMEM-High Glucose medium with characterized fetal bovine serum caused death of the cells. Thus, the type of fetal bovine serum seems to play an important role in the survival and growth of the yolk sac cells and may be it is related to the filtering process of these serums. In summary, the HyClone defined and characterized fetal bovine serums are filtered using 40 nm (0.04 µm) and 100 nm (0.1µm) pore-size filters, respectively. For mesenchymal stem cells from either human [25] or dog yolk sac [26] the authors demonstrated that ALPHA-MEM was the best medium to culture these cells, but they did not specify in detail regarding fetal bovine serum that was used in the culture. The culture of yolk sac explants resulted in a heterogeneous population that contained mainly fibroblast-like cells, but also a few epithelial-like cells. The latter must have been derived from the endothelial and mesothelium layers that characterized the inverted yolk sac of rodents including *Necromys* [31]; however, these cells died during the first cell passages. The fibroblast-like habits of the surviving cells [18] as well as the characteristic positive and negative responses to a set of surface markers clearly identified them as mesenchymal progenitor cells. This was apparently the first application of immunophenotyping markers in both flow cytometry and fluorescence, with consistent results. The cells were positive to mesenchymal stem cell markers (CD 73, 90, 105) and immunonegative to CD 34 and 45 as markers for hematopoetic stem cells. Thus, our cells had the characteristic pattern used to define mesenchymal stem cells in general [19]. They were also positive for Stro-1, a newer and probably better marker for such stem cells [32]. Interestingly, our fibroblast-like cells were immunopositive to CD 117 as a marker for haematopoetic cell precursors, suggesting that the cells still had the potential to give rise to hematopoietic lineages or vasculature components when using an induction medium. This was supported by the positive response to VEGF usually associated with development of the vascular system [33], [34]. Moreover, it is important to consider the importance of mesenchymal cells for hematopoiesis [35]. In addition, the positive response to PCNA fitted well into the observed growth and proliferation pattern. A similar pattern was reported for human mesenchymal stem cells [25]. Also, the cells were immunopositive to vimentin, cytokeratin and β-tubulin as markers for the cytoskeleton [36] and a high differentiation potential was indicated by the expression of the pluripotency markers Oct-3/4 and Nanog typical for progenitor cells [9], [37]. This pattern was consistent with previous results on yolk sac derived mesenchymal progenitor cells from the mouse, human and dog [22]–[26]. However, recent results from the human indicate that the exact effect of Oct4 is not fully understood [38], although it is regarded as a most critical regulator of pluripotency that regulates Nanog [39]. In addition, perhaps the differential potential of stem cells was due to a statistical property of cell populations instead of being exactly defined at the single-cell level [40].

The progenitor stem cells from the *Necromys* yolk sac were able to differentiated into adipogenic, osteogenic and chondrogenic lineages when using culture media with inducing factors [41]. A similar far-reaching differentiation potential was reported from mesenchymal progenitor cells of the yolk sac of the mouse [21], [22]. However, mesenchymal progenitor cells from other species had more limited potency. Adipogenic and osteogenic lineages were reported for the human, although the cells failed to differentiate into the chondrogenic lineage [24], [25]. Progenitor cells from the dog yolk sac differentiated into osteogenic and chondrogenic lineages only [26]. In contrast, human mesenchymal stem cells from adult tissues also had far-reaching differentiation potential [42]. Studies cited above indicate that the differentiation potential is not pluripotent and do not lead to cell lineages characteristic for all three germ layers, but to mesoderm-derived ones only, which is regarded as an indication for multipotency. Finally, based on the present and previous data [26] differentiation of the cell lineages from fetal membrane tissues such as the yolk sac required approximately 3 weeks, which was longer than differentiation assays derived from adult tissues [43]. Last but not least, our data confirmed that the cultured yolk sac derived progenitor cells did not cause development of tumors when transferred to a recipient, similar to other species [21], [22], [24]–[26].

## Conclusion

Mesenchymal stem cells with multipotent differentiation potential and sufficient expansion abilities were obtained from *Necromys* yolk sacs. The typical characteristics of mesenchymal progenitor cells from the mouse yolk sac seemed to be widespread among rodents; therefore, we inferred that such cells have a wide range of applications in regenerative medicine.
